# Variability in the Chemical Composition of a New Aromatic Plant *Artemisia balchanorum* in Southern Russia

**DOI:** 10.3390/plants11010006

**Published:** 2021-12-21

**Authors:** Gennadii V. Khodakov, Lavr A. Kryukov, Einat Shemesh-Mayer, Rina Kamenetsky-Goldstein

**Affiliations:** 1 V.I. Vernadsky Crimean Federal University, Agro-Technological Academy, 295007 Simferopol, Russia; gennadii-hodakov@mail.ru; 2 V.I. Vernadsky Crimean Federal University Center for Advanced Scientific and Technological Development, 295007 Simferopol, Russia; lavrkryukov@gmail.com; 3 Institute of Biology and Biomedicine, Lobachevsky State University of Nizhni Novgorod, 603022 Nizhni Novgorod, Russia; 4 The Agricultural Research Organization, Volcani Center, Institute of Plant Sciences, Rishon LeZion 7505101, Israel; shemeshe@volcani.agri.gov.il

**Keywords:** lemon wormwood, citral, linalool, geraniol

## Abstract

Lemon wormwood *Artemisia balchanorum* was recently introduced to southern Russia as a new aromatic plant. Based on biological and chemical characteristics, several populations with dominant citral, linalool, and geraniol production were selected for further development and maintained by seed propagation. Chemical analysis of five outstanding populations at three stages of annual development: vegetative, flower buds, and full flowering, confirmed that the seed populations retain the distinct dynamics of the dominant and minor components during the annual cycle and can be used for the commercial production of citral, linalool, and geraniol. Micropropagation in vitro allows for efficient clonal micropropagation and mass reproduction of elite cultivars and promising forms of *A. balchanorum* on a commercial scale but cannot serve as a source of direct and efficient production of secondary metabolites.

## 1. Introduction

Plants of the genus *Artemisia* L. (Asteraceae) have long been used in folk medicine and cuisine in many countries around the world. They contain biologically active substances that serve as the basis for the development of new drugs, some of which are already in use clinically [1,2]. In addition, *Artemisia* species have a high nutritional value. They are used as spices, seasonings, and aperitifs; as dyes for Chinese Qingtua dishes; for flavoring cakes, vinegars, alcoholic beverages, and tobacco; and consumed in the form of herbal tea and salads [3]. The biological significance of *Artemisia* stems from its complement of natural compounds: terpenoids (mainly monoterpenes of essential oils) and sesquiterpene lactones, flavonoids, lignans, alkaloids, steroids, phenolic acids, and coumarins [4,5], all of which are well known for a wide range of biological activities.

Similar to other aromatic plants, the most important chemical compounds of *Artemisia* for commercial production and for use in the cosmetic and pharmaceutical industries are essential oils [6]. Aromas, flavors, and medicinal benefits of the essential oils depend on their main organic compounds-terpenes [7,8]. In *Artemisia* spp., these include citral, with a strong citrus aroma; linalool, with a pleasant floral aroma and an antibacterial effect [9]; and geraniol, with a floral-fruit aroma and antibacterial and antifungal effects [10]. Citral is found in the oils of lemon myrtle, lemongrass, lemon tea tree, lime, and lemon. Citral is either a pair or a mixture of terpenoids with the molecular formula C_10_H_16_O. The two compounds are geometric isomers: the *E*-isomer is known as geranial (*trans*-citral), while the *Z*-isomer is known as neral (*cis*-citral). Linalool is a major constituent of the essential oils of coriander, cymbopogon, sweet orange flowers, lavender, and sweet basil, among others. Its odor is similar to floral, spicy wood and has a light citrus-like flavor. Geraniol is the primary component of the essential oils of citronella, rose, and palmarosa [11]. In addition to its pleasant aroma, geraniol exhibits insecticidal and repellent properties and is used as a pest control agent with low toxicity [12].

Synthesis and accumulation dynamics of aromatic compounds are regulated at different development stages by circadian rhythm and biological and abiotic factors [13,14]. The harvest stage can significantly affect the biochemical content. Therefore, from a practical point of view, the study of secondary metabolite composition and accumulation during the plant growth cycle will help to determine the optimal harvesting period for the specific compounds. When young annual stems are harvested in spring, this process eradicates flower development. At the same time, in many aromatic and medicinal plants, the best essential oils are accumulated at the stages of flower buds and full flowering [15,16]. Therefore, metabolite profiling of the chemical composition and oil content at different developmental stages is critical for the optimization of the harvest period for the desirable products.

Lemon wormwood (*Artemisia balchanorum* Krasch.) was introduced to the south of Russia from its natural habitats in the deserts and semi-deserts of Central Asia. Its chemical composition has been evaluated for cultivation as a desirable new aromatic crop and an alternative source for essential oils that are currently important to Russia. In Asia, this perennial semi-shrub with glaucous leaves and paniculate inflorescences is used in cooking and folk medicine [17]. The plants contain natural mono- and sesquiterpenes of essential oils, diterpene (phytol), triterpenes, and other useful compounds [18,19,20]. The selection for the specific biological and chemical traits in a large population of *A. balchanorum* has led to the development of several rather homogeneous lines with valuable qualities. These lines are currently maintained by seed propagation and can be used for commercial purposes. However, seed propagation of *A. balchanorum* is not always efficient due to a low rate of germination after seed storage. Propagation by lignified cuttings is used in commercial production, but the low (15–40%) survival rate of cuttings hinders the efficiency of agricultural production [21]. Difficulties inherent to traditional *A. balchanorum* breeding create problems preserving the genetic identity of promising genotypes when attempting to intensify the breeding process and enlarge the scale of replication. Therefore, in addition to the usual methods of reproduction of promising genotypes, attempts were made to propagate *A. balchanorum* using an in vitro culture method [22].

In this study, we identified and compared the chemical profiles of the selected seed populations of *A. balchanorum* at three stages of their annual development, from the vegetative stage in spring to full flowering in November. The results will be used in the future selection and propagation of new and promising *Artemisia* cultivars and to determine the optimal harvesting period for a high concentration of specific aromatic compounds.

## 2. Results and Discussion

The values of the mass fractions of the essential oils in terms of raw and dry weight for all plants studied during the spring shoot regrowth (Table 1) indicate that the most productive cultivars regarding the amount of essential oils are 136 and 150, with values of 1.43% (3.88%) and 1.64% (4.54%), respectively. Cultivar 210 yielded the lowest amount, 0.37% (1.08%). For oils distilled from plantlets of 130 obtained in vitro, the content of essential oil was low (0.04%).

Chromatographic analysis revealed more than 40 compounds, 16 of which were identified as myrcene, 1,8-cineole, α, γ-terpineol, αthujone, linalool, β-thujone, camphor, borneol, citronellal, neral (*cis*-citral), geraniol, geranial (*trans*-citral), oxycitronellal, geranyl acetate, and geranylpropionate. Among these, linalool, *cis*-citral, geraniol, *trans*-citral, and geranyl acetate are dominant (Tables S1 and S2).

**Figure 1:**
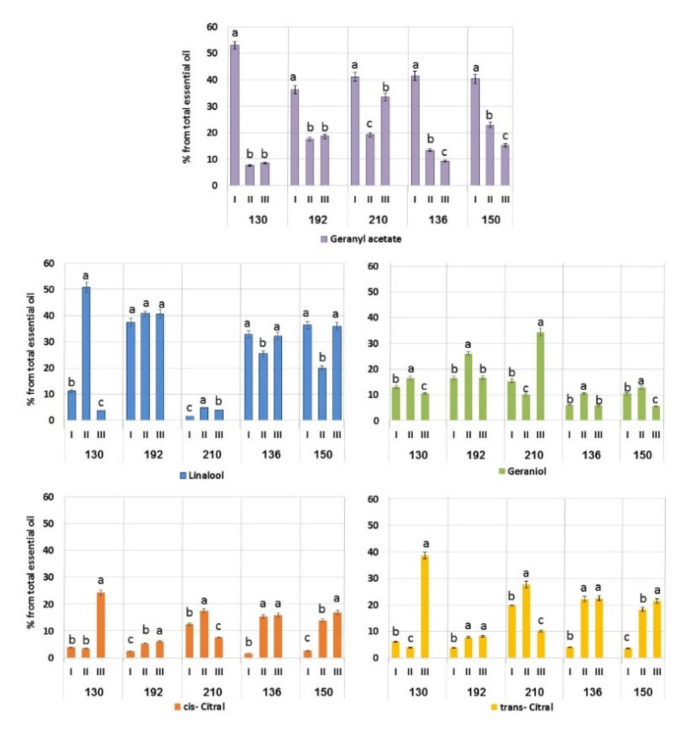
Accumulation of dominant components in the essential oil of cultivars 130, 192, 210, 136, and 150 of *A. balchanorum* at three stages of development, shown as a percentage from the total amount of essential oil. I—vegetative stage: during the spring regrowth at the end of May 2019, the entire annual shoots were sampled; II—flower buds: the tops of the stems with closed buds were sampled in mid-October 2019; III—flowering: the tops of the stems with inflorescences were sampled in mid-November 2019. Each dominant compound was analyzed separately in each cultivar for the differences between the stages. Bars represent standard deviation. One-way ANOVA and Student’s *t*-test were applied. Levels not connected by the same letter are significantly different (α = 0.05).

**Figure 2:**
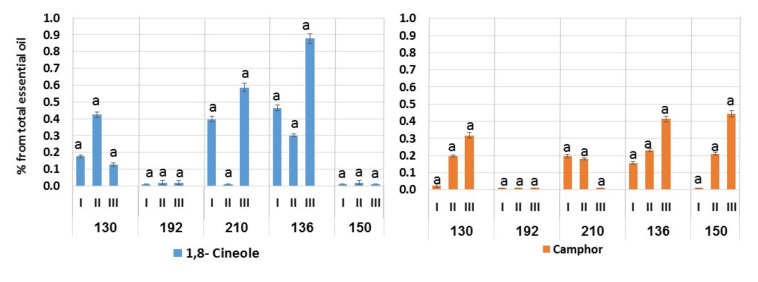
Dynamics of accumulation of valuable minor components in the essential oil of plants in the cultivars of *A. balchanorum* 130, 192, 210, 136, and 150. I—vegetative stage: during the spring regrowth at the end of May 2019, the entire annual shoots were sampled; II—flower buds: the tops of the stems with closed buds were sampled in mid-October 2019; III—flowering: the tops of the stems with inflorescences were sampled in mid-November 2019. Each compound was analyzed separately in each cultivar for the differences between the stages. Bars represent standard deviation. One-way ANOVA and Student’s *t*-test were applied. Levels not connected by the same letter are significantly different (α = 0.05).

A comparative analysis of essential oils of all cultivars in three developmental stages is shown in Figure 1 and Figure 2. Most of the compounds showed significant statistical interaction between the cultivar and the developmental stage (Table S3). In other aromatic plants, e.g., *Cymbopogon* spp. [23], esterification processes are prevalent in the tops of the shoots during spring regrowth. Biosynthetic chains from geraniol are acetylated with acetyltransferase and then de-acetylated to yield geranyl acetate. In *A. balchanorum*, while geranyl acetate was found at all stages of plant development, it predominated in the vegetative stages (Figure 1). For instance, in cultivar 130, geranyl acetate comprised more than 50% of the main essential oils observed during the vegetative stage, while linalool became dominant at the flower bud stage (58%) and citrals (63%) during flowering. Geranyl acetate dominates during the first stage in all cultivars, but at the stages of flower buds and flowering, geraniol dominated in 192 and 210, while citrals increased in 130, 210 136, and 150.

Differences between stages in each cultivar were also observed in the presence of minor economically important components–1,8-cineol and camphor (Figure 2).

We found that cultivar 130 can serve as a rich source of natural citrals, a valuable product, which can be extracted directly from plant material at the flowering stage. Similarly, *Artemisia santonica* f. citralifera has been proposed as an efficient producer of natural citrals, with the highest content (44.6%) found during the flowering stage [24]. These lemon-scented monoterpenes are largely used in food and cosmetics, and *A. balchanorum* can serve as an additional source for their efficient extraction. Linalool (3.6%) and geraniol (10.6%) were found in the cultivar 130 during the flowering stage, along with a significant amount of geranyl acetate (8.5%). The sharp increase in the content of citrals during flowering (Figure 1) indicates weakened esterification and increased oxidation due to the activation of geranyl dehydrogenase [25].

Another valuable compound, linalool, can be extracted from this cultivar in the flower bud stage when its content reaches 50% of the total amount of essential oils. Linalool possesses a comprehensive range of bioactive properties, which can be exploited for pharmaceutic and cosmetic applications [26]. In addition to these major compounds, cultivar 130 contains the valuable minor components 1,8-cineole and camphor (Figure 2). The content of 1,8-cineole, which increases from 0.18% during spring regrowth to 0.42% at the flower bud stage, decreases to 0.13% during the mass flowering period. The content of camphor during the growing season gradually increases from 0.03% to 0.32%.

Since cultivar 130 has such an impressive potential for natural terpene production, we performed analysis of its microcuttings propagated in vitro. However, the results were rather different from the analysis of the adult plants. Only low content of essential oils (0.04%) enriched with three components: geranyl acetate (70%) and two citrals (combined content of 25%) were found in 20-day-old microcuttings. Linalool and geraniol were not detected. The dominant presence of geranyl acetate is explained by the fact that geraniol and its acetate are precursors of citral [23]. Similarly, in the adult plant, geranyl acetate is predominant during vegetative development in the spring (Figure 1). We conclude, therefore, that in vitro microcuttings remain at a very early stage of development and biosynthesis of secondary metabolites. They are not suitable for the production of essential oils but can be used for fast and efficient micropropagation. After hardening and acclimation, microcuttings will develop into adult plants, which will then serve for essential oil production. Further research is needed to establish an efficient in vitro system as the basis for commercial propagation of elite varieties of *A. balchanorum* that would allow continuous production of plant material under field conditions yielding a significant quantity of desirable compounds.

Cultivar 192 contains rather stable amounts of linalool, which remains stable during the entire growing season, from spring to late fall (Figure 1). The amount of citrals increases slightly from the vegetative to reproductive stages, while the content of geraniol peaks only in the flower bud stage to 26%. The content of geranyl acetate is highest during spring regrowth (36.2%) and then decreases to 17–18%. This cultivar contains only trace amounts of 1,8-cineole and camphor (Figure 2).

The essential oil obtained from cultivar 210 during the mass flowering period was high in geraniol content (34.3%) (Figure 1). In the vegetative plants and prior to flowering, the geraniol content was relatively low and then increased sharply during the mass flowering period. An increase in the content of geraniol during the flowering could be caused by decreased activity of geranyl dehydrogenase as proposed for *Cymbopogon* [23]. This line is characterized by a low linalool content but contains the valuable minor components 1,8-cineole and camphor (Figure 2).

We conclude that the seed populations of cultivars 192 and 210 contain significant amounts of linalool and geraniol, respectively, and that seed propagation can be used for the stable commercial reproduction of these lines. Two additional cultivars, 136 and 150, did not produce any dominant compounds. Their linalool content, depending on the growing stage, ranged from 20% to 36%. The accumulation of citrals in both cultivars increased during the growing season, while the content of geranyl acetate decreased (Figure 1). The amount of geraniol was relatively low and ranged from 5.6% to 13% during the growing season. Both lines contain 1,8-cineole and camphor. In cultivar 136, the content of 1,8-cineole decreased from 0.5% to 0.3% during the transition from the spring regrowth to the flower bud stage and then increased to 0.9% during the mass flowering period. In both lines, the camphor content increased during the growing season (Figure 2). Thus, the two cultivars with the highest oil production share similar dynamics of accumulation of dominant components and minor substances.

In general, the synthesis and accumulation of secondary metabolites are very complex processes, affected by genetic, developmental, and environmental factors [14]. Thus, the samples from Central Asia contained higher amounts of 1,8-cineole (29.9%), α-thujone (11.7%), and camphor (11.2%), but low quantities of linalool and citrals [27]. Studies by Svidenko and Rabotyagov [28] of *A. balchanorum* and *A. taurica* and their hybrids also show extremely high variation in the major secondary metabolites. Our analysis of the composition and dynamics of essential oils during the growing season of *A. balchanorum* confirms at least three choices for the natural production of the essential oils. Under our experimental conditions, cultivar 130 contains high amounts of citrals, while linalool dominates in cultivar 192 and geraniol in cultivar 210.

In agricultural practice, the outstanding selections of *Artemisia* are propagated either by seeds or vegetatively. However, neither system is not consistent. For example, seed propagation of *A. annua* for artemisinin extraction is employed on commercial plantations [29,30], but low seed viability and germination rate impair commercial propagation. Therefore, the selection of the outstanding cultivars in the specific location should be accompanied by efficient true-to-type propagation.

Micropropagation techniques were studied in different *Artemisia* species. Both tissue culture-regenerated plants and rooted cuttings of *A. annua*, *A. nilagirica*, and *A. japonica* performed better than plants derived from seeds in terms of uniformity, yield, and biochemical content [29,31,32]. The use of in vitro technology to produce plants of *A. balchanorum* with homogeneously high essential oils will certainly improve the quality and yield of the commercial products extracted from this species but will not allow direct extraction of the desired compounds from in microcuttings.

Finally, the question of how best to extract and separate pure compounds and select for special chemotypes of lemon wormwood corresponds with the concept of the “entourage effect”, better known from cannabis studies [33,34]. This effect refers to the synergistic mode of multiple compounds, which may potentiate pharmaceutical efficacy. In this context, *Artemisia* could be an interesting model in which to study the synergistic effects of various essential oils in aromatic plants.

## 3. Materials and Methods

### 3.1. Plant Material and Micropropagation

**Figure 3:**
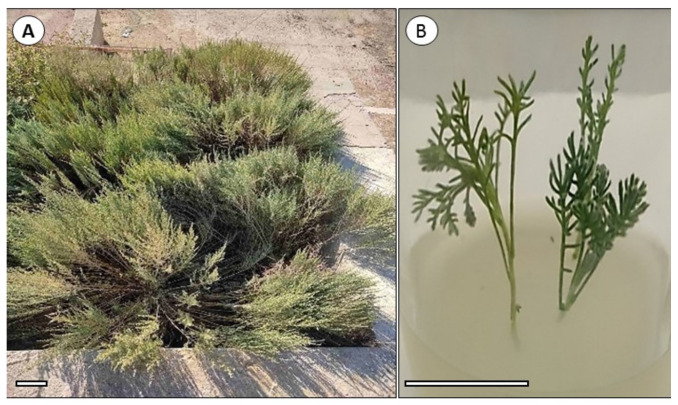
Plants of *Artemisia balchanorum*, cultivar 130; (**A**) 3-year-old plant in October 2019 (bar = 10 cm), (**B**) microcutting in vitro (bar = 5 cm).

The selected lines of *A. balchanorum* (cultivars 130, 192, 210, 136, and 150) were obtained from the Nikita Botanical Garden (Yalta, Russia) and maintained vegetatively in the living collection (Figure 3A). In November 2016, one-seeded achene fruits were collected from each line and sown in soil immediately after harvest. Material from the 3-year-old offspring of each seed population became the source for the qualitative and quantitative analysis of the essential oils. The constituents of the essential oils in these five cultivars were studied during the 2019 growing season at three developmental stages: (1) vegetative stage: during the spring regrowth at the end of May 2019, the entire annual shoots were sampled; (2) flower buds: the tops of the stems with closed buds were sampled in mid-October 2019 (Figure 3A); and (3) flowering: the tops of the stems with inflorescences were sampled in mid-November 2019. Each sample of approximately 300 g contained the tops of annual stems from 6 to 9 plants of each cultivar. Plant tops were mixed and divided into three equal parts, then 5 g were taken from each replicate and dried to determine moisture content and dried weight. The rest of the samples were weighed and used to extract essential oil.

Cultivar 130 was propagated in vitro according to a protocol proposed by Mitrofanova et al. [22]. Seeds were sterilized in 70% alcohol for 30–40 s, treated with a 0.1% solution of diacid for 10 min, and washed 4 times in sterile distilled water (for 10 min each time). The seeds were planted in test tubes on MS medium and placed in a dark chamber for 72 h. The test tubes were then transferred to a chamber at 25 °C with a relative humidity of 70%, illumination of 5000–8000 lux, and a photoperiod of 16 h. Young plants emerged after 1.5–2 months. Normally developed seedlings were propagated using microcuttings, i.e., the shoots were aseptically dissected into fragments about 1 cm in length and transferred to fresh MS medium for rooting and growth. After 25–30 days, well-developed cuttings were obtained, ready for the next cycle (Figure 3B). Twenty-day-old microcuttings were used for the extraction of essential oils.

### 3.2. Chemical Analysis

Essential oils from the adult plants and microcuttings were extracted by hydro-distillation of freshly harvested raw materials with petroleum ether, followed by recalculation of the mass fraction of the oil by raw and dry weight. Hydro-distillation was carried out with a reflux condenser, and the essential oil was collected in a Ginsberg trap. Component identification was carried out by comparison with standards (Merck, Germany); n-hexane was the zero mark.

The oil of each replicate was separated into components to determine its qualitative and quantitative composition. The average value of the total number of components in the essential oil was calculated. The separation of essential oils into components was carried out using standard gas-liquid chromatography on a model 3700 chromatograph (St.-Petersburg, Russia) on a 50 m long quartz capillary column (SE-52 phase; ID, 0.25 mm; carrier gas, nitrogen; flow rate, μL/min). The temperature of the evaporator and detector was 220 °C. The initial temperature of the thermostat was 80 °C, which increased to 170 °C following a subsequent increase of 3 °C/min for 30 min. Identification of components was carried out by comparison to internal standards. Sampling was carried out in triplicate for each growing season, and essential oils were obtained from each repetition.

### 3.3. Statistical Analysis

One-way and two-way ANOVA, as well as Student’s *t*-test (α = 0.05) analyses, were applied, using JMP-Pro, Statistical Discovery, version 15, SAS Institute.

## 4. Conclusions

Three distinct cultivars of *A. balchanorum* with a high content of citral, linalool, and geraniol were found as valuable sources for natural essential oils. The seed populations of these cultivars maintain distinct dynamics of the dominant and valuable minor components during annual development. Further selection and efficient clonal micropropagation will allow mass reproduction of valuable cultivars and promising forms of *A. balchanorum* on a commercial scale.

The combined effect of the secondary metabolites (“entourage effect”) can be more effective in the whole composition rather than in single components. Further development of agronomical systems and technologies for uniform propagation will allow for the use of valuable compounds as insect repellents and anti-antimicrobial, as well as in cosmetics and the food industry.

## Figures and Tables

**Table 1:** Percentage of the fraction of essential oils in fresh and dry samples of five lines of *A. balchanorum* (vegetative stage). One-way ANOVA and Student’s *t*-test analyses were applied. Levels not connected by the same letter are significantly different (α = 0.05).

Cultivar Number	Content in Fresh Mass (%)	Dry Weight (%)	Content in Dry Mass (%)
130	0.65 d	34.03 a	1.91 d
192	1.17 c	34.61 a	3.38 c
210	0.37 e	34.26 a	1.08 e
136	1.43 b	36.86 a	3.88 b
150	1.64 a	36.12 a	4.54 a

## Supplementary Materials

The following are available online at https://www.mdpi.com/article/10.3390/plants11010006/s1; Table S1. Composition of essential oil (%) in *A. balchanorum* cultivar 130 at various stages of development and in vitro propagated plantlets. I, vegetative; II, flower buds; III, full flowering; Table S2. Composition of the essential oils (%) in the offspring of four cultivars of *A. balchanorum* at three developmental stages: I—vegetative; II—flower buds; III—full flowering; Table S3. Analysis of variances by Two-Way ANOVA for five dominant and two minor compounds of *Artemisia balchanorum* essential oil. Most compounds show significant statistical interaction between the cultivar and the developmental stage (α = 0.05).
