# Supplementary material for: Variability in the Chemical Composition of a New Aromatic Plant Artemisia balchanorum in Southern Russia
Source: Plants (Basel). 2021 Dec 21;11(1):6. doi: 10.3390/plants11010006 (PMC8747407; doi:10.3390/plants11010006)
Supplement: Supplementary file 1 [file plants-11-00006-s001.zip › plants-1450074-supplementary.pdf]

Supplementary Table S1. Composition of essential oil (%) in *A. balchanorum* cultivar 130 at various stages of development and *in vitro* propagated plantlets. I, vegetative; II, flower buds; III, full flowering. (+) – only traces detected; (-) – not detected

#	Compound	**Offspring population**			***In vitro***
		I	II	III	Plantlets
1	Myrcene	0.17 ±0.01	0.09 ±0.004	0.03	+
2	1,8-Cineole	0.18±0.01	0.42 ±0.02	0.13 ±0.01	+
3	γ-Terpineol	0.08±0.003	0.41 ±0.02	+	+
4	α-Thujone	+	0.04	0.58±0.03	+
5	Linalool	11.2±0.6	50.8 ±2.52	3.66 ±0.18	+
6	β-Thujone	-	-	0.03	-
7	Camphor	0.03	0.2±0.01	0.32 ±0.02	+
8	Borneol	0.99±0.05	+	0.38±0.02	+
9	Citronellal	3.79±0.18	14.1 ±0.7	0.75 ±0.04	+
10	α-Terpineol	+	0.14 ±0.01	0.43 ±0.02	-
11	*cis*-Citral	3.81±0.19	3.14 ±0.15	24.26 ±1.21	25.0±1.21*
12	Geraniol	13.08±0.65	16.45 ±0.82	10.61± 0.52	+
13	*trans*-Citral	6.13±0.31	3.88 ±0.19	38.68 ±1.92	+
14	Oxycitral	+	0.97 ±0.05	0.03	+
15	Geranil acetate	53.09±2.65	7.59 ±0.38	8.46 ±0.42	70.0 ±3.44
16	Geranil propionate	0.22 ±0.01	0.46 ±0.02	1.14 ±0.06	+

*Total content of *cis*-Citral and *trans*-Citral.

Supplementary Table S2. Composition of the essential oils (%) in the offspring of four cultivars of *A. balchanorum* at three developmental stages: I, vegetative; II, flower buds; III, full flowering. (+) – only traces detected; (-) – not detected

#	Cultivar	192			210			136			150		
	**Compound**	**I**	**II**	**III**	**I**	**II**	**III**	**I**	**II**	**III**	**I**	**II**	**III**
1	Myrcene	0.24 ±0.01	0.3±0.01	0.71±0.03	0.57 ±0.03	0.37 ±0.02	0.27 ±0.01	+	0.29 ±0.01	0.46 ±0.02	-	0.34±0.02	+
2	1,8-Cineole	+	0.04	0.04	0.4 ±0.02	+	0.59 ±0.03	0.46 ±0.02	0.3 ±0.01	0.87 ±0.04	+	0.04	+
3	γ-Terpineol	0.27 ±0.01	0.32±0.02	0.22±0.01	0.41 ±0.02	0.64 ±0.03	0.3 ±0.01	0.39 ±0.02	0.31 ±0.01	0.51 ±0.02	0.27 ±0.01	0.15±0.01	0.33 ±0.02
4	α-Thujone	+	0.03	0.05	0.24 ±0.01	+	2.3 ±0.1	0.73 ±0.04	0.3 ±0.01	0.32 ±0.02	0.4 ±0.02	+	0.03
5	Linalool	37.44 ±1.87	40.7±2.03	40.6 ±2.02	1.47 ±0.07	4.8 ±0.2	3.89 ±0.19	32.8 ±1.61	25.54 ±1.28	32.12 ±1.60	36.33±1.82	20.0 ±0.9	35.98 ±1.79
6	β-Thujone	-	+	+	0.07 ±0.003	0.23 ±0.01	+	+	0.24 ±0.01	0.36 ±0.02	-	+	0.38 ±0.02
7	Camphor	+	+	+	0.2 ±0.01	0.18 ±0.01	+	0.16 ±0.01	0.23 ±0.01	0.41 ±0.02	+	0.21 ±0.01	0.44 ±0.02
8	Borneol	+	0.63 ±0.03	0.82±0.04	0.27 ±0.01	+	0.61 ±0.03	0.03	0.62 ±0.02	0.36 ±0.02	-	+	+
9	Citronellal	1.74 ±0.08	+	+	1.55 ±0.08	0.78 ±0.04	0.24 ±0.01	4.0 ±0.19	0.21 ±0.01	+	3.13 ±0.15	1.45±0.07	0.49 ±0.02
10	α-Terpineol	+	0.41 ±0.02	0.24 ±0.01	0.33 ±0.02	0.63 ±0.03	0.21 ±0.01	0.37 ±0.02	0.92 ±0.04	1.0 ±0.04	0.29 ±0.01	0.33±0.02	+
11	*cis*-Citral	2.41 ±0.12	5.31 ±0.26	6.03±0.30	12.49 ±0.62	17.47±0.87	7.48 ±0.37	1.63 ±0.08	15.5±0.8	15.92 ±0.79	2.62 ±0.11	13.97±0.69	16.87 ±0.84
12	Geraniol	16.5 ±0.82	26.04 ±1.29	16.69±0.83	15.38 ±0.76	10.2 ±0.51	34.3 ±1.71	6.07 ±0.30	10.59±0.52	5.79 ±0.29	10.5 ±0.52	12.98±0.64	5.62 ±0.28
13	*trans*-Citral	3.71 ±0.18	7.74 ±0.38	8.04±0.40	19.8 ±1	27.74±1.38	10.05 ±0.50	3.18 ±0.16	22.23±1.11	22.45 ±1.12	3.56 ±0.18	18.15±0.90	21.49 ±1.07
14	Oxycitral	+	+	0.67±0.03	-	0.47 ±0.02	-	+	0.03	+	0.05	0.08±0.003	+
15	Geranil acetate	36.23 ±1.81	17.63 ±0.87	18.6±0.9	41.13 ±2.05	19.25±0.96	33.51 ±1.67	41.5 ±2.06	13.42±0.66	9.26 ±0.46	40.27±2.01	22.9±1.14	15.24 ±0.76
16	Geranil propionate	0.35 ±0.02	0.44 ±0.02	0.44±0.02	1.58 ±0.08	1.07 ±0.05	1.3 ±0.06	0.47 ±0.02	1.43 ±0.07	0.8 ±0.04	0.49 ±0.02	1.37±0.07	0.53 ±0.02

Supplementary Table S3. Analysis of variances by Two-way ANOVA for five dominant and two minor compounds of *Artemisia balchanorum* essential oil. Most compounds show significant statistical interaction between the cultivar and the developmental stage (α=0.05).

**Geranyl acetate** **Source**	**DF**	**Sum of Squares**	**Mean Square**	**F Ratio**	**Prob > F**
Model	14	6351.06	453.65		
Error	30	2633.35	87.78		
C. Total	44	8984.41		5.17	<0.0001
Cultivar	4	839.40		2.39	0.07
Stage	2	4911.70		27.98	<0.0001
Cultivar*Stage	8	740.97		1.06	0.42

Linalool

Source	DF	Sum of Squares	Mean Square	F Ratio	Prob > F
Model	14	9307.23	664.80		
Error	30	1944.81	64.83		
C. Total	44	11252.04		10.26	<0.0001
Cultivar	4	6574.96		25.36	<0.0001
Stage	2	184.40		1.42	0.26
Cultivar*Stage	8	2470.45		4.76	0.0008

trans-Citral

Source	DF	Sum of Squares	Mean Square	F Ratio	Prob > F
Model	14	4925.83	351.85		
Error	30	23.93	0.80		
C. Total	44	4949.76		441.04	<0.0001
Cultivar	4	805.11		252.30	<0.0001
Stage	2	1392.86		872.97	<0.0001
Cultivar*Stage	8	2354.79		368.97	<0.0001

Geraniol

Source	DF	Sum of Squares	Mean Square	F Ratio	Prob > F
Model	14	2506.35	179.03		
Error	30	26.18	0.87		
C. Total	44	2532.53		205.13	<0.0001
Cultivar	4	1173.93		336.27	<0.0001
Stage	2	63.32		36.27	<0.0001
Cultivar*Stage	8	1281.09		183.48	<0.0001

cis-Citral

Source	DF	Sum of Squares	Mean Square	F Ratio	Prob > F
Model	14	2116.89	151.21		
Error	30	11.72	0.39		
C. Total	44	2128.61		387.19	<0.0001
Cultivar	4	343.43		219.86	<0.0001
Stage	2	742.45		950.60	<0.0001
Cultivar*Stage	8	885.92		283.57	<0.0001

1,8- Cineole

Source	DF	Sum of Squares	Mean Square	F Ratio	Prob > F
Model	14	2.95	0.21		
Error	30	0.09	0.003		
C. Total	44	3.04		71.21	<0.0001
Cultivar	4	1.84		155.11	<0.0001
Stage	2	0.19		32.63	<0.0001
Cultivar*Stage	8	0.81		34.03	<0.0001

Camphor

Source	DF	Sum of Squares	Mean Square	F Ratio	Prob > F
Model	14	0.90	0.06		
Error	30	0.04	0.001		
C. Total	44	0.94		48.75	<0.0001
Cultivar	4	0.36		68.05	<0.0001
Stage	2	0.17		63.06	<0.0001
Cultivar*Stage	8	0.37		35.21	<0.0001
